# Bibliographical—the American Medical Temperance Quarterly

**Published:** 1893-10

**Authors:** 


### Bibliographical—The American Medical Temperance Quarterly.

At the meeting of the American Medical Association held in Washington, D. C., in 1891, the American Medical Temperance Association was organized.

The object of this association is to study and investigate the action of alcohol, as both a beverage and a medicine. The alcoholic problem has reached such proportions, and has become a subject of such intense interest in all political, social and religious circles as to demand scientific study.

This association has in its membership many of the leading physicians of the United States, nearly, if not quite all of whom have discarded the use of alcoholic stimulants in the practice of medicine. Foremost in this matter stands the venerable Dr. N. S. Davis, of Chicago, Ill., who for nearly forty years has discarded the use of alcohol in medical practice, and such an array of facts and arguments is he able to produce as to carry conviction to the mind of almost every unprejudiced person.

This association holds its annual meeting at the time and place of the meeting of the American Medical Association, so that two meetings have been held, one at Detroit and the other at Milwaukee, since the organization, and at each meeting the membership was increased.

Such importance has this association assumed, that it was determined at the last meeting to establish a journal for promoting the interest of the society and the cause which it espouses. In response to that resolution the first number of the American Medical Temperance Quarterly was issued, July 1893. Its Editorial Committee consists of Drs. N. S. Davis, A.M., M.D., LL.D., of Chicago, T. D. Crothers, A.M., M.D., of Hartford, Conn., and J. H. Kellogg, M.D., of Battle Creek, Mich.

The present number is an exceedingly interesting one, discussing many phases of the alcoholic question, especially in reference to its medical uses. The question is one of intense interest to every one practicing medicine in any of its branches, and nowhere, perhaps, will be found focalized on this question more direct thought, facts and arguments than will be presented in this journal. It should be in the hands of every medical man, whether he be specialist or general practitioner, all are interested in the question, whatever their personal views may be in regard to it.

It consists of twenty-four large, closely printed pages, at subscription rate of fifty cents per annum. Subscriptions may be sent to Dr. J. H. Kellogg, M.D., Battle Creek, Mich.

